# Effects of *Lactiplantibacillus plantarum*, combined with konjac flour or lignocellulose, on silage quality and bacterial diversity of high-moisture forage rape silage

**DOI:** 10.1128/spectrum.03072-25

**Published:** 2026-03-23

**Authors:** Encun Du, Na Zhao, Wei Zhu, Wanzheng Guo, Jintao Wei

**Affiliations:** 1 Hubei Key Laboratory of Animal Embryo and Molecular Breeding, Institute of Animal Science and Veterinary Medicine, Hubei Academy of Agricultural Sciences 117996, Wuhan, China; 2 Xianning Animal Husbandry Development Center, Xianning, China; Institute of Microbiology, Chinese Academy of Sciences, Beijing, China

**Keywords:** aerobic stability, cost-effective additive, fermentation quality, high-moisture silage, *Lactobacillus*

## Abstract

The combination of *Lactiplantibacillus plantarum* (LP) and konjac flour (KF) were found to improve the silage quality of high-moisture forage rape previously. To identify a cost-effective alternative to KF, we further investigated the effects of the combination of LP and lignocellulose (LC) on the aerobic stability, fermentation quality, chemical composition, and bacterial community of high-moisture forage rape silage. After 60 days of ensiling, the combination of LP and LC improved the aerobic stability, dropped the pH value, increased the lactic acid content, and decreased the butyric acid content in ensiled forage rape (*P* < 0.05), which were similar to the effects of the LP and KF combination. Both combinations decreased the content of crude protein (*P* < 0.05) while increasing the content of neutral detergent fiber in forage rape silage (*P* < 0.05). Moreover, LP combined with KF or LC exerted a notable influence on the bacterial community, as evidenced by reduced Shannon and Simpson indices and absolute dominance of *Lactobacillus* (*P* < 0.05). In conclusion, the combination of LP and LC could be used as a cost-effective alternative to the LP and KF combination and enhance the silage quality of high-moisture forage rape through modulating its bacterial community.

**IMPORTANCE:**

Applying additives to high-moisture forage rape silage is critical for guaranteeing its fermentation quality. Both KF and LC exhibit high water-holding and water-binding capacities, with LC being more cost-effective. Our previous study demonstrated enhanced silage quality of high-moisture forage rape by LP+KF. In the present study, we report for the first time the effect of LP+LC on improving the silage quality of high-moisture forage rape, which was similar to that of LP+KF. The results indicate that the combination of LP and LC effectively enhances the abundance of *Lactobacillus* while reducing the abundance of undesirable bacteria, such as enterobacteria and clostridia, thereby effectively improving the silage quality of forage rape. This study provides a valuable reference for the development of novel silage additives for high-moisture forage rape and further contributes to alleviating the shortage of high-quality forage in China.

## INTRODUCTION

Forage rape (*Brassica napus*), which is also known as rapeseed, oilseed rape, or canola, is the second-largest oil-producing crop grown worldwide. In addition to oil production, forage rape has become part of the ruminant grazing system in Europe, Australia, and New Zealand (1), since the biomass yield of forage rape is greater than 40 ton fresh matter/hm^2^ and its nutritional value was quite high (2). In China, there is a long-term shortage of high-quality forage, especially in southern China, which challenges the development of ruminant breeding. The high-humidity climatic conditions and acidic soil conditions in southern China are extremely detrimental to the growth of alfalfa, but they are conducive to the growth of forage rape. Therefore, the efficient utilization of forage rape in ruminant production is one of the important solutions to alleviate the shortage of high-quality forage (3).

Due to its seasonal harvest, forage rape should be properly preserved to provide a continuous supply. Ensiling is a traditional technology for preserving green forage, which can extend storage time, improve palatability, and supply year-round availability of moist forage. However, it is hard to make high-quality silage since forage rape has high-moisture content (>800 g/kg fresh matter) and high buffering capacity (4). Generally, the most economical and practical approach for moisture management is field wilting. Given the moist and rainy weather during forage rape harvest in southern China (from late winter to the following spring), it is not practical to reduce moisture content by field wilting. The use of absorbent during ensiling has been suggested as an alternative to wilting and has been reported to improve the quality of silage (5). The fermentation quality of silage mainly depends on the microbial community and its metabolites. Our previous study found that the combination of *Lactiplantibacillus plantarum* (LP) and konjac flour (KF) improves the aerobic stability and fermentation quality of high-moisture forage rape by increasing the abundance of *Lactobacillus* greatly and decreasing the abundance of enterobacteria and clostridia (6).

KF, processed from the root of konjac tuber (*Amorphophallus konjac*), mainly contains konjac glucomannan (KGM) and displays high water-holding and water-binding capacities (7). Currently, KF or KGM is commonly utilized as food additive or dietary supplement to support the health of humans and animals (8), both of which command relatively high prices. Although the combined use of KF and LP exerts a favorable effect on the silage of high-moisture forage rape (6), the high cost of KF could potentially restrict its application in silage production.

Lignocellulose (LC), or plant dry matter, is a constituent of plant cell walls, mainly composed of cellulose, hemicellulose, and lignin. As the most abundant and prevalent renewable biomass on earth, the global production of LC is approximately 200 billion tons per year (9), making it easily obtainable and inexpensive. Similar to KF, LC also had high water-holding and water-binding capacities (10); thus, we hypothesized that LC might be used as an alternative to replace KF, and the combination of LP and LC may benefit the ensiling of high-moisture forage rape. Therefore, the present study was conducted to compare the effects of the LP and KF combination with the LP and LC combination on aerobic stability, fermentation quality, chemical composition, and bacterial community of high-moisture forage rape silage.

## MATERIALS AND METHODS

### Materials and silage preparation

The forage rape (variety Huayouza 62) was harvested from the experimental field of the Hubei Academy of Agricultural Sciences (Wuhan, China, 114°01′E to 114°35′E and 29°58′N to 30°32′N) at the flowering stage, when the crop was in full bloom. Prior to ensiling, the harvested materials were chopped to 2–3 cm length with a crop chopper (CZ009, Shengxiang Co., Linyi, China) and then allocated to the following ensiling treatments: control (no additive, CK), LP+KF (5 × 10^6^ cfu/g of LP and 30 g/kg of KF based on fresh matter), and LP+LC (5 × 10^6^ cfu/g of LP and 30 g/kg of LC based on fresh matter). The LP was purchased from Guangzhou Weiyuan Biotechnology Co., Ltd., and its viable count is 1 × 10^11^ cfu/g. The KF used in the current study contained 882 g/kg of dry matter (DM), and 610 g/kg of KGM and 80 g/kg of crude protein (CP) based on DM. The LC used in the current study was made from fresh wood and contained 879 g/kg of DM and 950 g/kg of total dietary fiber based on DM.

In detail, 600 g of fresh forage rape was blended with the corresponding amounts of additives: 30 mg LP plus either 18 g KF or 18 g LC. The LP inoculant was prepared in 5 mL of normal saline solution and sprayed onto the fresh forage rape. An equivalent amount of the normal saline solution was sprayed onto the fresh forage rape in the CK group. Then, the mixture was packed into plastic silo bags and sealed using a vacuum sealer (VS5500, Aperts, Dongguan, China). Each bag contained 600 g of fresh forage rape and the corresponding amounts of additives. A total of 9 silage bags (3 treatments with 3 replicates per treatment) were prepared and stored in dark conditions at an ambient temperature of 15–30°C. After 60 days of ensiling, aerobic stability, fermentation property, chemical composition, and microbial community were analyzed.

### Assessment of aerobic stability

To assess the aerobic stability of the silos after 60 days of ensiling, the temperature was measured by inserting a digital thermometer (TP-101, Honeywell Co., NC, USA) into the geometric center of the silage mass. According to He et al. (11), approximately 400 g of silages were placed loosely into a clean plastic bucket, and two layers of cheesecloths were placed over each container to decrease moisture volatilization and potential contamination. The buckets were stored in polystyrene boxes to inhibit fast heat diffusion. Aerobic stability is the time for which silage is stable before its temperature rose 2°C above room temperature (12).

### Analysis of fermentation property and chemical composition

To determine the fermentation profile, 10 g of silage samples were mixed well with 90 mL of sterile water and then filtered through four layers of cheesecloth. The filtrate was immediately subjected to pH measurement and organic acid quantification. The pH value of the filtrate was promptly analyzed using a pH meter. A high-performance liquid chromatography (HPLC, LC-20A, Shimadzu, Japan) with a 210 nm UV detector and a Shodex RSpak KC-811S-DVB gel C column (8.0 mm × 30 cm; Shimadzu, Tokyo, Japan) quantified the levels of organic acids (including lactic acid, acetic acid, propionic acid, isobutyric acid, and butyric acid). A 3 mmol/L HClO_4_ solution was used as the eluent at 50°C, with a flow rate of 1.0 mL/min.

A sample of fresh forage rape prior to ensiling was dried at 65°C in a forced-air oven for 48 h to determine the DM content, after which it was ground to pass a 1.0 mm screen for chemical analysis. About 20 g of silage samples were collected from each silo bag after 60 days of ensiling to analyze the chemical composition. Standard procedures of the Association of Official Analytical Chemists (13) were used to examine the contents of CP, ether extract (EE), and crude ash. The contents of neutral detergent fiber (NDF) and acid detergent fiber (ADF) were analyzed following the method of Van Soest et al. (14). The content of water-soluble carbohydrates (WSC) was analyzed using the anthrone method (15).

### Bacterial diversity analysis

Total DNA was extracted using the E.Z.N.A. Bacterial DNA Kit (Omega Biotek, Norcross, GA, USA) according to the manufacturer’s protocol. Then, the concentration of DNA was determined using spectrophotometry, and its quality was evaluated using 2% agarose gel electrophoresis. The microbial 16s rDNA was amplified using the hypervariable V4 region PCR primers (515F: 5′-GTGCCAGCMGCCGCGG-3′ and 806R: 5′-GGACTACHVGGGTWTCTAAT-3′) with a unique error-correcting barcode for each sample.

After purification and quantification, the PCR products were sequenced using the Illumina platform (Genewiz Co. Ltd., Suzhou, China). Then, high-quality clean tags were obtained using the QIIME quality-control process (Version 1.9.1), and the chimera sequences were detected and removed using the UCHIME algorithm. Then, the effective sequences were aligned into operational taxonomic units (OTUs) analysis using the software VSEARCH (version 1.9.6) based on 97% sequence similarity. Alpha diversity was analyzed by the metrics of Chao1, Ace, Shannon, and Simpson. Principal coordinate (PCoA) and hierarchical clustering analyses based on Bray-Curtis distance of OTUs were performed to represent the beta diversity and the similarity of bacterial community structures among different silage treatment groups. The representative OTU sequences were then compared with the Silva 132 database using the Ribosomal Database Program Classifier for taxonomic classification (at 80% confidence threshold) at the kingdom, phylum, class, order, family, and genus levels.

### Statistical analysis

Results are given as mean values and pooled standard errors. One-way ANOVA and Tukey’s multiple comparisons were used to analyze the data. Data were considered significant when *P* < 0.05. All statistical procedures were performed using SPSS version 22.0 (IBM Inc., Chicago, IL, USA). The linear discriminant analysis effect size (LEfSe) method was utilized to determine the differentially abundant taxonomies among different treatments, with the default threshold linear discriminant analysis (LDA) score of 4. Metastats analysis was performed on the normalized OTU relative abundance data at the genus level to identify significantly differentially abundant genera, with FDR correction applied to *P*-values and significance defined as adjusted *P* < 0.05.

## RESULTS

### Chemical composition of fresh forage rape before ensiling

The average chemical composition of fresh forage rape was analyzed prior to ensiling (Table 1). Results showed that the DM content of fresh forage rape was 144.0 g/kg. Based on DM, the CP, NDF, and ADF contents were 144.5, 490.2, and 325.7 g/kg, respectively. The WSC, EE, and crude ash contents were 75.6, 47.3, and 95.1 g/kg based on DM, respectively.

**TABLE 1:** Chemical characteristics of fresh forage rape prior to ensiling (±SD, *n* = 3)^*a*^

Item	Forage rape
Dry matter (g/kg FM)	144.0 ± 14.7
Crude protein (g/kg DM)	144.5 ± 6.6
Neutral detergent fiber (g/kg DM)	490.2 ± 11.9
Acid detergent fiber (g/kg DM)	325.7 ± 10.4
Water soluble carbohydrate (g/kg DM)	75.6 ± 5.3
Ether extract (g/kg DM)	47.3 ± 9.4
Crude ash (g/kg DM)	95.1 ± 7.8

^*a*^ DM, dry matter; FM, fresh matter; SD, standard deviation.

### Aerobic stability of forage rape silage

**Fig 1:**
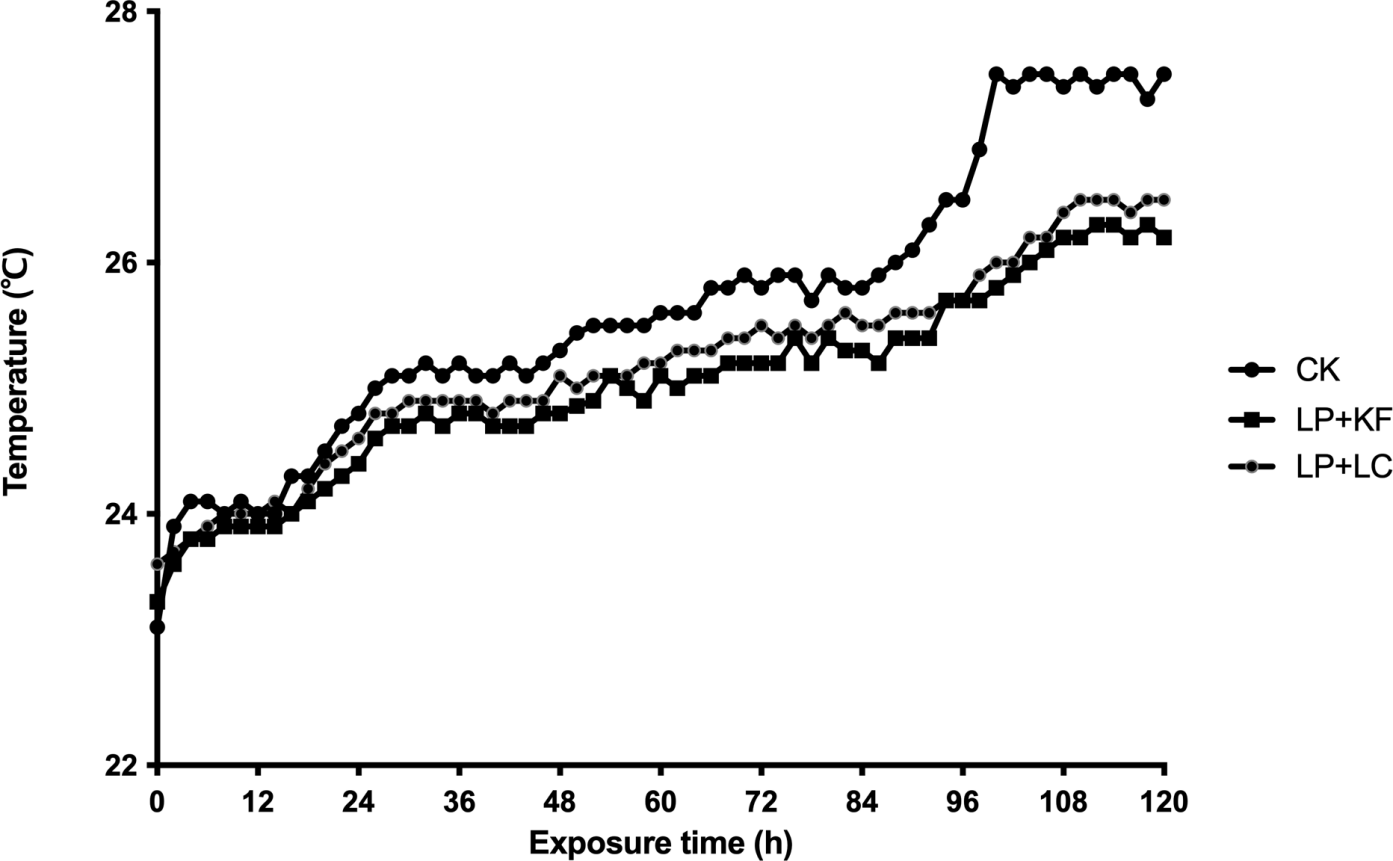
The temperature dynamics of forage rape silage during the aerobic exposure. CK, the control; LP+KF, *Lactiplantibacillus plantarum* + konjac flour; LP+LC, *Lactiplantibacillus plantarum* + lignocellulose.

The aerobic stability of forage rape silage is displayed in Fig. 1 and Table 2. After an aerobic exposure period of 28.0 h, silage in the control group (no additive, CK) deteriorated. During the continuous monitoring time of 120 h, CK reached its maximum temperature of 27.5°C after an aerobic exposure period of 100 h. Compared with CK, the combination of LP and KF (LP+KF) and the combination of LP and LC (LP+LC) significantly improved the aerobic stability of forage rape silage (*P* < 0.05). The maximum temperature caused by secondary fermentation was higher in group CK than in LP+KF and LP+LC (*P* < 0.05), and the time to reach the maximum temperature was shorter in CK (*P* < 0.05). The temperature dynamics of forage rape silage treated with LP+KF and LP+LC were similar, and the two treatment groups displayed similar aerobic stability (*P* > 0.05).

**TABLE 2:** Aerobic stability of forage rape silage^a^

Treatment	CK	LP+KF	LP+LC	SEM	*P-*value
Aerobic stability (h)	28^b^	76^a^	83^a^	9	<0.001
Maximum temperature (°C)	27.50^a^	26.27^c^	26.47^b^	0.19	<0.001
Time to reach maximum temperature (h)	100^b^	111^a^	111^a^	2	<0.001

^a^ CK, the control; LP+KF, *Lactiplantibacillus plantarum* + konjac flour; LP+LC, *Lactiplantibacillus plantarum* + lignocellulose; SEM, pooled standard error; means with different superscripts in the same row differ significantly (*P* < 0.05).

### Fermentation property and chemical composition of ensiled forage rape

**Fig 2:**
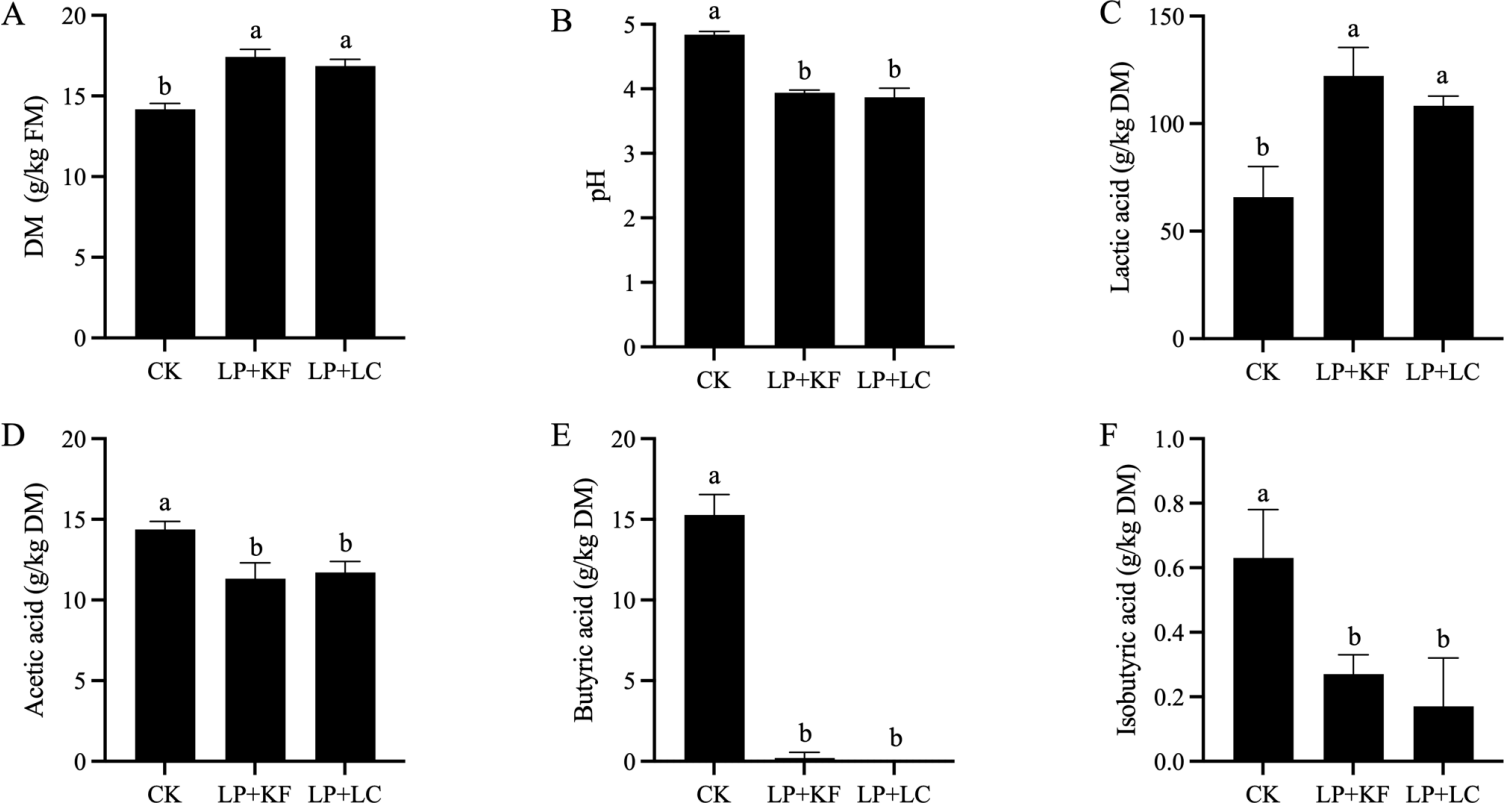
Fermentation property of ensiled forage rape: DM content (**A**), pH valuee (**B**), and contents of lactic acid (**C**), acetic acid (**D**), butyric acid (**E**) and isobutyric acid (**F**). CK, the control; LP+KF, *Lactiplantibacillus plantarum* + konjac flour; LP+LC, *Lactiplantibacillus plantarum* + lignocellulose; DM, dry matter; FM, fresh matter. Columns with different superscripts differ significantly (*P* < 0.05).

The pH value and organic acid contents of forage rape silage are illustrated in Fig. 2. The pH value dramatically decreased in LP+KF and LP+LC (*P* < 0.05), and their contents of lactic acid were close to or more than two times that of CK (*P* < 0.05). However, there was a slight decrease in the content of acetic acid in LP+KF and LP+LC (*P* < 0.05). In the present study, the content of butyric acid in group CK was 15.27 g/kg DM, which decreased to a very low level in the treated groups, even becoming undetectable (*P* < 0.05). In addition, less isobutyric acid was found in the treated groups (*P* < 0.05). The fermentation properties of forage rape ensiled with LP+KF and LP+LC were similar (*P* > 0.05), and the production of butyric acid and isobutyric acid was numerically lower in LP+LC.

The chemical composition of forage rape silage is shown in Table 3. Compared with CK, lower CP content and higher NDF content were found in group LP+KF and LP+LC (*P* < 0.05), and higher ADF content was also found in LP+LC (*P* < 0.05). In contrast with LP+KF, the treatment of LP+LC decreased the content of crude ash (*P* < 0.05), while increasing the contents of NDF and ADF (*P* < 0.05).

**TABLE 3:** Chemical composition of forage rape silage^a^

Item	CK	LP+KF	LP+LC	SEM	*P-*value
CP (g/kg DM)	146.29^a^	117.45^b^	118.82^b^	4.75	<0.001
NDF (g/kg DM)	501.15^c^	550.16^b^	584.90^a^	12.58	<0.001
ADF (g/kg DM)	341.64^b^	344.28^b^	395.78^a^	8.9	<0.001
Crude ash (g/kg DM)	96.55^ab^	102.55^a^	94.24^b^	1.44	0.017

^a^ DM, dry matter; CP, crude protein; NDF, neutral detergent fiber; ADF, acid detergent fiber; CK, the control; LP+KF, *Lactiplantibacillus plantarum* + konjac flour; LP+LC, *Lactiplantibacillus plantarum* + lignocellulose; SEM, pooled standard error; means with different superscripts in the same row differ significantly (*P* < 0.05).

### Bacterial community of forage rape silage

In the current study, a coverage value of 0.999 to 1.000 was observed in all silage samples, indicating that almost all bacteria were identified. The alpha diversity of the bacterial community is shown in Table 4. Although Ace and Chao1 indices were not influenced by the treatments (*P* > 0.05), both Shannon and Simpson indices decreased significantly (*P* < 0.05). In addition, the Simpson index of LP+LC was even lower than LP+KF (*P* < 0.05).

**TABLE 4:** Alpha diversity of the bacterial community of forage rape silage^a^

Item	CK	LP+KF	LP+LC	SEM	*P*-value
Ace	67.64	54.75	45.21	4.31	0.081
Chao1	66.21	52.41	43.71	4.23	0.064
Shannon	3.42^a^	0.90^b^	0.41^b^	0.47	<0.001
Simpson	0.86^a^	0.24^b^	0.09^c^	0.12	<0.001

^a^ CK, the control; LP+KF, *Lactiplantibacillus plantarum* + konjac flour; LP+LC, *Lactiplantibacillus plantarum* + lignocellulose; SEM, pooled standard error; means with different superscripts in the same row differ significantly (*P* < 0.05).

**Fig 3:**
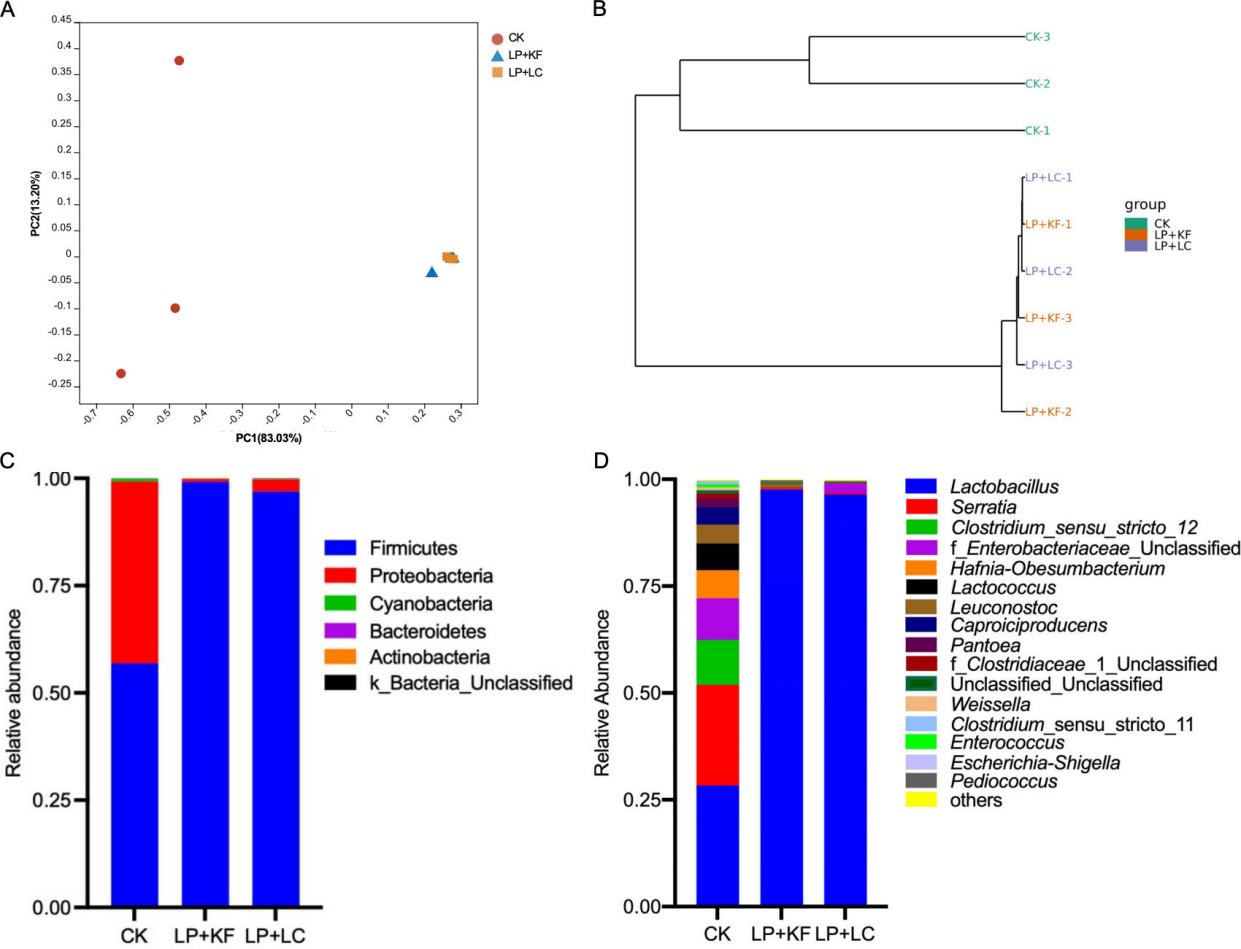
PCoA (**A**) and hierarchical (**B**) clustering of the bacterial community of ensiled forage rape. The bacterial community and abundances at the phylum (**C**) and genus (**D**) levels. CK, the control; LP+KF, *Lactiplantibacillus plantarum* + konjac flour; LP+LC, *Lactiplantibacillus plantarum* + lignocellulose.

According to the PCoA analysis (Fig. 3A), the bacterial communities were distinctly clustered by the treatment group. Notably, the microbial community structures of the LP+KF and LP+LC groups showed a nearly complete overlap. The principal coordinates 1 and 2 accounted for 83.05% and 13.2% of the total variation, respectively. Consistent with the PCoA analysis results, the hierarchical clustering tree showed that the bacterial communities of the LP+KF and LP+LC groups clustered into one branch, while those of the CK group formed a separate branch (Fig. 3B).

Regarding the bacterial community composition, Firmicutes and Proteobacteria were the two dominant bacteria in the CK group (Fig. 3C). With the addition of LP+KF or LP+LC, the bacterial community was dramatically changed. In both the LP+KF and LP+LC groups, the relative abundance of Firmicutes was elevated to over 95%, while that of Proteobacteria was reduced to an extremely low level. At the genus level, *Lactobacillus* and *Serratia* were predominant in group CK, accounting for 28.32% and 23.59%, respectively (Fig. 3D). The subdominant bacteria in CK were *Clostridium* _sensu _stricto _12 and f_*Enterobacteriaceae*_Unclassified, accounting for 10.52% and 9.71%, respectively. The treatment of LP+KF and LP+LC elevated the relative abundance of *Lactobacillus* greatly, accounting for 97.54% and 96.43%, respectively. Meanwhile, the relative abundances of *Serratia*, f_*Enterobacteriaceae*_Unclassified, and *Clostridium*_sensu_stricto_12 were markedly decreased.

**Fig 4:**
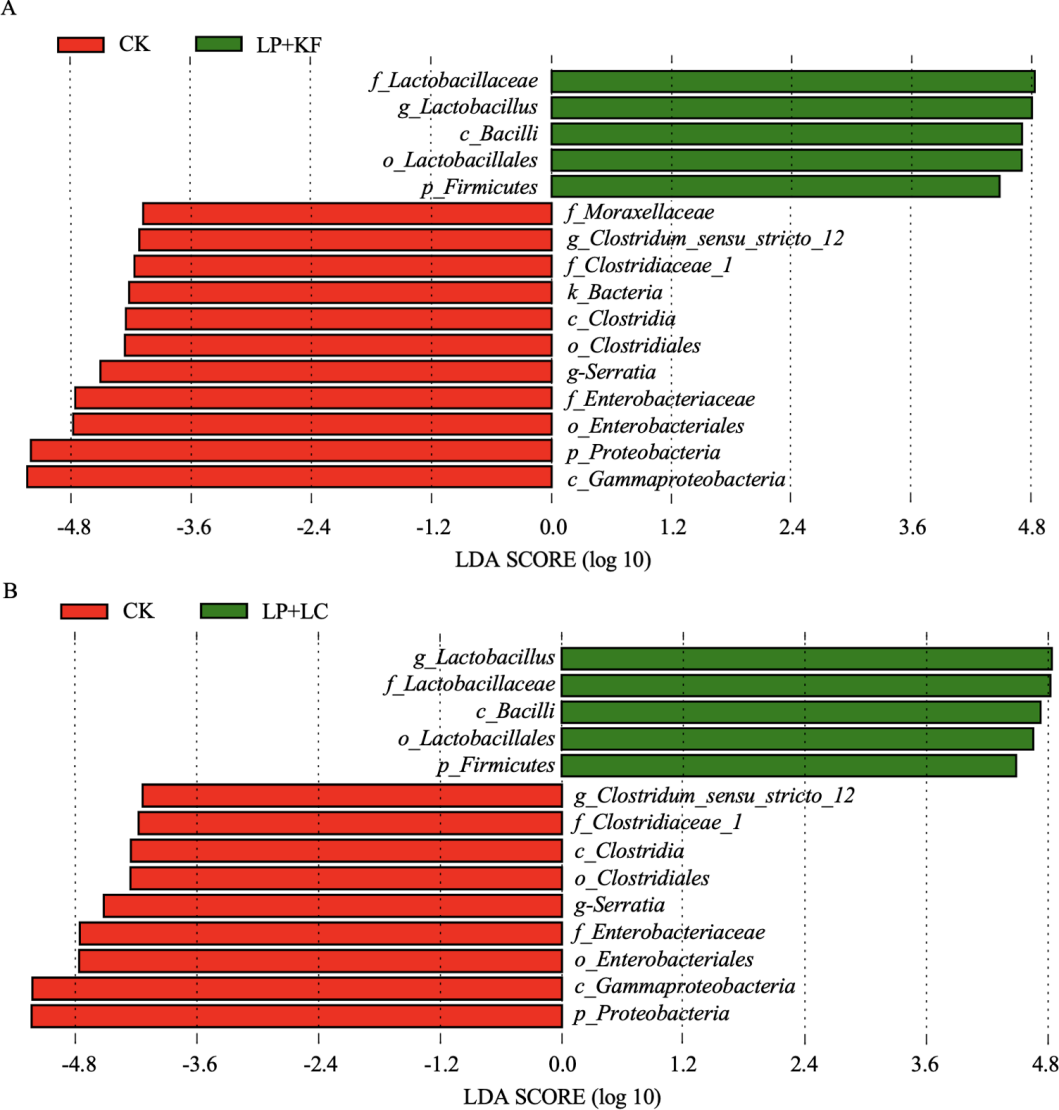
Comparison of bacterial variations using LEfSe analysis: CK vs. LP+KF (**A**) and CK vs. LP+LC (**B**). CK, the control; LP+KF, *Lactiplantibacillus plantarum* + konjac flour; LP+LC, *Lactiplantibacillus plantarum* + lignocellulose.

The LEfSe method was utilized to assess the differences in microbial community and explore the specific bacteria in each group (LDA score > 4). According to Fig. 4A, Proteobacteria was the most abundant phylum, and Moraxellaceae, Clostridiaceae_1, and Enterobacteriaceae were the most abundant families, while *Serratia* and *Clostridium*_sensu_stricto_12 were the most abundant genera in CK. In contrast, Firmicutes and *Lactobacillus* (from order to genus) were the most abundant bacteria in LP+KF, which could serve as biomarkers of LP+KF. Based on Fig. 4B, the differences in microbial community between CK and LP+LC were almost the same with those between CK and LP+KF. Besides, no difference was observed between LP+KF and LP+LC according to the LEfSe analysis (LDA score > 4), indicating similar bacterial communities between the two treatment groups.

**Fig 5:**
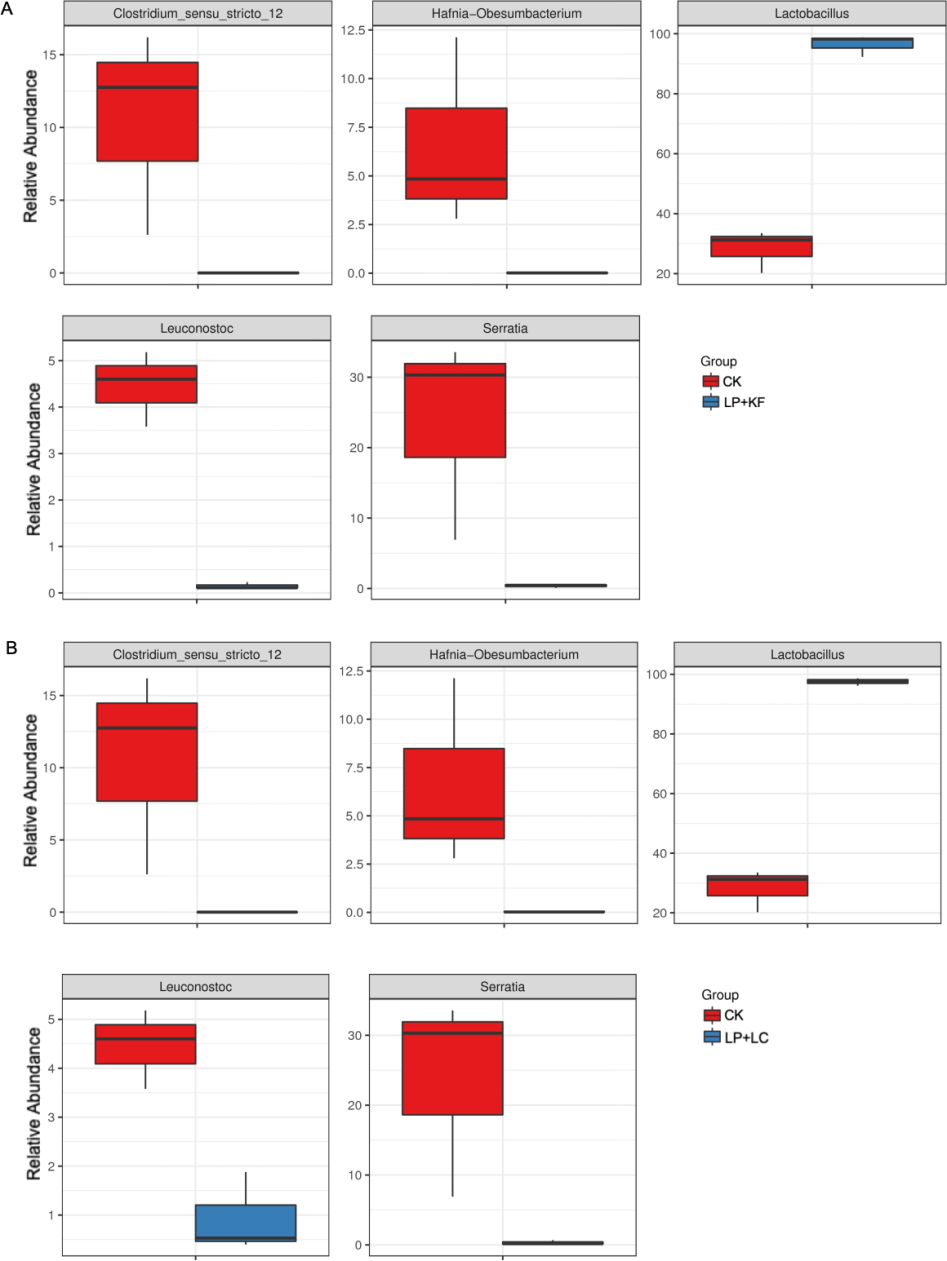
Comparison of bacterial variations using Metastats analysis: CK vs. LP+KF (**A**) and CK vs. LP+LC (**B**). CK, the control; LP+KF, *Lactiplantibacillus plantarum* + konjac flour; LP+LC, *Lactiplantibacillus plantarum* + lignocellulose.

According to the Metastats analysis, the LP+KF and LP+LC groups exhibited significantly lower relative abundances of *Clostridium*_sensu_stricto_12, *Hafnia-Obesumbacterium*, *Leuconostoc*, and *Serratia* (adjusted *P* < 0.05), and a significantly higher relative abundance of *Lactobacillus* (adjusted *P* < 0.05) compared with CK (Fig. 5). There was no difference in the relative abundance of bacterium at genus level between LP+KF and LP+LC (adjusted *P* > 0.05).

## DISCUSSION

During ensiling, epiphytic lactic acid bacteria (LAB) ferment WSC to lactic acid, thereby lowering the pH value and inhibiting the growth of spoilage microorganisms in the ensiled material (16, 17). The WSC content of forage rape is 75.6 g/kg DM, which exceeds the theoretical requirement (60–70 g/kg DM) for achieving well-preserved silage (18). However, when moisture content exceeds 700 g/kg DM, it becomes challenging to produce high-quality silage as the excessive moisture dilutes the population of LAB and counteracts the pH drop (17, 19). In addition, under high-moisture conditions, ensiling can lead to the proliferation of harmful microorganisms, such as *Clostridium* species, which compete with LAB and can break down and utilize the proteins in forage, resulting in high DM loss, extensive proteolysis, excessive accumulation of biogenic amines, ammonia, and butyric acid, as well as poor feed palatability (20, 21). Notably, the moisture content of fresh forage rape was over 800 g/kg. Thus, measures should be taken to improve the fermentation quality of forage rape. Our previous study demonstrated that both the inoculation of LP and the addition of KF enhance the silage quality of fresh forage rape, with their combination exerting a synergistic effect (6).

Aerobic deterioration of silage during the feed-out phase is a significant problem for farm profitability and feed quality worldwide, which has a negative influence on nutrition preservation and feed intake by animals (20, 22). Generally, aerobic deterioration of silage is sponsored by yeasts, which metabolize lactic acid and WSC into CO_2_ and water under aerobic conditions. Based on our previous study, the combination of LP and KF improved the aerobic stability of forage rape silage, but the addition of LP or KF alone did not exhibit any improvement (6). In the current study, the combination of LP, either with KF or LC, effectively improved the aerobic stability of forage rape silage and displayed similar temperature dynamics during the aerobic exposure period. This implies that LC can replace KF and plays a synergistic role with LP in improving the aerobic stability of forage rape silage, which may be related to the similar bacterial community and metabolites between the two combinations (discussed below).

The structure of LC is complex and stable, consisting of 40–50% cellulose, 25–30% hemicellulose, 15–20% lignin, and traces of pectin, nitrogen compounds, and inorganic ingredients (23, 24). Although LC can be degraded by rumen microbes, it is poorly digested by the gut microbiota of non-ruminant mammals and chickens. Hence, most efforts have been made in the pretreatment and enzymatic hydrolysis of LC in feedstuff to improve its digestibility (25). In recent years, LC has been recognized as an innovative insoluble dietary fiber with remarkable hydration capacity. Studies in farm and companion animals showed that dietary addition of LC may have potential effects on digestive physiology and function (26, 27), especially in alleviating constipation and shortening farrowing time of pregnant sows (28, 29). However, the effect of LC as a silage additive has not been reported yet.

Silage pH plays an important role in the evaluation of fermentation property, and silage with pH 4.2 or lower is considered well-fermented and ensures better aerobic stability (19). In the present study, the pH value of the naturally ensiled forage rape silage was 4.84, which was much higher than the benchmark pH of 4.20, indicating poor fermentation quality. In contrast, the pH value of the additive-treated silage was below 4.00, which ensures good preservation of high-moisture forage rape silage. As the main metabolite, lactic acid is another important evaluation index of fermentation quality. According to the current study, the combination of LP, either with KF or LC, doubled the production of lactic acid, which was the main reason for the significant pH reduction of forage rape silage. Moreover, the content of butyric acid in both treatment groups decreased to a very low level. Butyric acid is undesirable in silage as its generation is an energy-waste metabolism (19). When butyric acid exceeds 5 g/kg DM, it indicates substantial clostridial activity and will impair livestock feed intake (19). In this study, the decreased butyric acid content in the treated silage might be due to inhibited clostridial fermentation and decreased abundance of clostridia according to the 16s rDNA sequencing. Based on the present study, the fermentation property between LP+LC and LP+KF was similar, although LC mainly consisted of insoluble fiber and KF mainly consisted of soluble fiber. It could be inferred that in terms of improving the ensiling quality of high-moisture forage rape, the hydration capacity of additives is more important than fiber type.

In the present study, the LP+KF and LP+LC groups contained less CP content. This could be explained by the lower CP content of KF and LC compared with forage rape. On the contrary, the LP+KF and LP+LC groups had higher NDF content, and this may be due to the higher NDF content of KF and LC than forage rape. Also, the alteration of microbial activities might result in the difference in nutrient preservation. Based on the current study, the ensiled forage rape of LP+LC contained higher amounts of NDF and ADF than LP+KF. The divergence might be related to the different types of fiber between LC and KF. LC contained more structural fiber than KF, and this probably impacted the degradation effect of LP on NDF and ADF during ensiling. Based on the fermentation property and chemical composition, LC could be used as an additive to substitute for KF and improve the ensiling quality, as well as preserve the nutrition of high-moisture forage rape when combined with LP.

According to the current study, the combination of LP, either with KF or LC, significantly decreased the Shannon and Simpson indices. The Shannon and Simpson indices were used to represent the community diversity. This result implied decreased bacterial diversity, which may be due to the predominance of *Lactobacillus* and the inhibited growth of undesirable bacteria in the treated silage. Similar results have also been found by Zhao et al. (17) and Yang et al. (30), where the inoculation of LAB increased the abundance of *Lactobacillus* and decreased the bacterial diversity, resulting in enhanced silage quality of high-moisture alfalfa silage.

Normally, as the major bacterial strain with desirable functions, *Lactobacillus* is dominant in well-preserved forage silage because it is responsible for lactic acid fermentation, pH reduction, and silage spoilage prevention during ensiling (16, 17). In the untreated forage rape silage, *Lactobacillus* accounted for only 28.32%, while *Serratia* and *Clostridium*_sensu_stricto_12 were the most abundant genera, and Enterobacteriaceae was the most abundant family based on the LEfSe analysis. *Serratia* is a genus of facultatively anaerobic bacteria of Enterobacterium. Although the role of *Serratia* during ensiling is still unclear, members of *Serratia* have been found to cause important infections in humans, animals, and insects (31). Clostridia and enterobacteria are undesirable in silage as they cause proteolysis and secondary butyric fermentation enhancing ammonia and biogenic amine production (32, 33). Biogenic amines formed in the early stage of ensiling are probably due to decarboxylation of amino acids by enterobacteria during the initial aerobic phase, whereas those formed later in the ensiling period are the result of subsequent growth of proteolytic clostridia (19). Biogenic amines are potentially toxic, as many of these putrefaction-associated compounds are malodorous and unpalatable, reducing feed intake in livestock and depressing milk production in cattle (34).

The treatments of both LP+KF and LP+LC, however, promoted the abundance of *Lactobacillus*, which accounted for more than 95% of the total population. It could be attributed to the inoculation of LP, which directly increased the population of *Lactobacillus*. Moreover, the addition of KF and LC with high hydration capacity was expected to decrease the water activity, support the pH drop of high-moisture forage rape, and inhibit undesirable microbes, which might be also conducive to the growth of *Lactobacillus*. In the present study, the relative abundance of enterobacteria was reduced, and clostridia was almost eliminated in the treated silage. In addition, the relative abundances of genera *Clostridium*_sensu_stricto_12, *Hafnia-Obesumbacterium*, *Leuconostoc*, and *Serratia* were significantly decreased in the treated silage based on the Metastats analysis. *Clostridium* spp. are known to thrive in silage with high pH value (>4.50), high-moisture content (>70%), and high buffering capacity (35). Ensiling methods that cause a rapid and sufficient drop in silage pH will help to prevent the activity of enterobacteria and clostridia (19). The combination of LP, either with KF or LC, increased the content of lactic acid and promoted the pH drop during fermentation, thereby inhibiting the abundance of enterobacteria and clostridia. *Hafnia-Obesumbacterium* and *Serratia* were found in spoiled chicken breast, and these bacteria were effectively inhibited by some antimicrobial substance from *Lactobacillus* (36). Mixed silage of faba bean with oat improved fermentation quality, showing a higher relative abundance of *Lactobacillus* and lower relative abundances of *Hafnia-Obesumbacterium* and *Serratia* (37), which is similar to the present study. These results suggest that these bacteria were acid intolerant. Alternatively, the dominance of *Lactobacillus* took advantage of the competition with them for fermentation substrates, resulting in their lower relative abundance. *Leuconostocs* is a kind of heterofermentative LAB, which is generally found living in association with plant material and dairy products (38). During silage fermentation, *Leuconostocs* grows vigorously in the early stage of ensiling and ferments WSC to produce D-lactic acid, CO_2_, and acetic acid. The inoculation of *Leuconostocs* was found to cause fermentation loss and did not improve the silage quality of alfalfa and Italian ryegrass (38). In the present study, the relative abundance of *Leuconostocs* was decreased in the treated silages after 60 days of ensiling, which may be attributed to the competitive inhibition exerted by *Lactobacillus*. According to the similar bacterial community between LP+KF and LP+LC, it could be speculated that LC could be used as an additive to substitute for KF and improve the silage bacterial community when added with the combination of LP.

Based on market quotations for feed additives, the current unit price of LC ranges from 1 to 10 RMB/kg, whereas that of KF is approximately 60 to 80 RMB/kg. In the present study, both additives were applied at an identical dosage of 30 g/kg of fresh forage rape. According to this application rate, the cost of LC per ton of ensiled forage rape is calculated to be 30–300 RMB, accounting for merely 1.2–17% of the cost incurred by KF. This clearly indicates that LC has a significant economic advantage over KF and its combined utilization with LP represents a cost-effective strategy for improving the silage quality of high-moisture forage rape.

In the future, further studies, including *in vitro* digestion experiments and *in vivo* trials, are needed to comprehensively investigate the effects of LP+LC on the nutrient degradability, palatability, and feeding value of high-moisture ensiled forage rape, thereby providing technical support for its large-scale production and industrial application.

### Conclusion

LP combined with LC improved aerobic stability, increased lactic acid content, decreased butyric acid content, and lowered pH value of high-moisture forage rape, which was similar to the effects of the combination of LP and KF. LP combined with LC also increased NDF and ADF contents. Moreover, both LP+KF and LP+LC remarkably enriched the abundance of *Lactobacillus* and inhibited the abundance of undesirable bacteria, such as enterobacteria and clostridia. These results proved that the combination of LP and LC could be used as a cost-effective alternative to the LP and KF combination and enhance the silage quality of high-moisture forage rape by modulating its bacterial community.

## Data Availability

The data sets presented in this study can be found in online repositories. The names of the repository/repositories and accession number can be found here: https://catalog.data.gov/dataset/sequence-read-archive-sra, PRJNA1048652.
